# The Metabolic Syndrome, Inflammation, and Colorectal Cancer Risk: An Evaluation of Large Panels of Plasma Protein Markers Using Repeated, Prediagnostic Samples

**DOI:** 10.1155/2017/4803156

**Published:** 2017-03-22

**Authors:** Sophia Harlid, Robin Myte, Bethany Van Guelpen

**Affiliations:** Department of Radiation Sciences, Oncology, Umeå University, Umeå, Sweden

## Abstract

Metabolic syndrome (MetS), a set of metabolic risk factors including obesity, dysglycemia, and dyslipidemia, is associated with increased colorectal cancer (CRC) risk. A putative biological mechanism is chronic, low-grade inflammation, both a feature of MetS and a CRC risk factor. However, excess body fat also induces a proinflammatory state and increases CRC risk. In order to explore the relationship between MetS, body size, inflammation, and CRC, we studied large panels of inflammatory and cancer biomarkers. We included 138 participants from the Västerbotten Intervention Programme with repeated sampling occasions, 10 years apart. Plasma samples were analyzed for 178 protein markers by proximity extension assay. To identify associations between plasma protein levels and MetS components, linear mixed models were fitted for each protein. Twelve proteins were associated with at least one MetS component, six of which were associated with MetS score. MetS alone was not related to any protein. Instead, BMI displayed by far the strongest associations with the biomarkers. One of the 12 MetS score-related proteins (FGF-21), also associated with BMI, was associated with an increased CRC risk (OR 1.71, 95% CI 1.19–2.47). We conclude that overweight and obesity, acting through both inflammation and other mechanisms, likely explain the MetS-CRC connection.

## 1. Introduction

Metabolic syndrome (MetS) is associated with numerous adverse health outcomes, such as cardiovascular disease (CVD), type 2 diabetes mellitus (T2DM), and several types of cancer [1–4]. It comprises metabolic abnormalities including central obesity, hypertension, dysglycemia, and dyslipidemia [5, 6]. The western lifestyle, with a diet high in fat and simple carbohydrates combined with low physical activity, likely contributes to the current rise in incidence [7].

One hallmark of MetS is a state of chronic inflammation. Several previous studies have reported protein biomarkers associated with MetS [8], with a large proportion directly related to inflammation, for example, tumor necrosis factor alpha (TNF-*α*) and interleukin 6 (IL-6). Visceral adiposity appears to play a central role in driving the inflammatory state, as this type of adipose tissue secretes monocyte chemoattractant protein-1 (MCP-1) and proinflammatory cytokines, which in turn induce macrophage infiltration of the tissue [9]. The chronic, low-grade inflammation resulting from visceral adiposity may therefore be a major mechanism behind the established association between MetS and colorectal cancer (CRC) [10, 11]. However, whether this connection is dependent on actual MetS development, or is solely an artefact of obesity, remains to be elucidated.

We hypothesized that the connection between MetS and CRC is driven by inflammation, with body composition as an important component, and that inflammatory proteins associated with MetS would therefore also associate with CRC risk. Using a unique collection of repeated samples from the Västerbotten Intervention Programme (VIP) in northern Sweden, we analyzed large panels of inflammatory and cancer biomarkers in relation to MetS and its components. The MetS-related biomarkers identified were examined in relation to the risk of developing CRC.

## 2. Materials and Methods

### 2.1. Study Population

All study participants were selected from the Västerbotten Intervention Programme (VIP), initiated in 1985 and still ongoing [12]. All residents of the county are invited to a general health exam at 10-year intervals (starting at 40 years). They also donate a blood sample and fill out an extensive questionnaire on health and lifestyle.

Samples for this study were originally selected as part of a prospective study of CRC biomarkers. All CRC cases had to have a verified CRC diagnosis within five years after the latest sampling (excluding samples collected within three months of diagnosis) and have at least two available blood samples in the biobank. All but one case set had samples collected ten years apart. We selected an equal number of control subjects, matched on age (±12 months), sex, and sampling dates (±12 months). Controls had to be cancer-free at the latest follow-up (Dec. 31, 2014). For both cases and controls, only samples collected after at least eight hours of fasting were included, and none of the samples had previously been thawed. After all inclusions and exclusions, the study included repeated samples from 69 prospective CRC cases and 69 matched controls, resulting in 276 samples analyzed.

The project was approved by the Regional Ethical Review Board of Umeå University, Sweden. All VIP participants provide a written informed consent before donating their samples for research purposes, and they retain the right to withdraw that consent at any time in the future.

### 2.2. MetS Variables

MetS components were measured as body mass index (BMI), triglyceride levels, total cholesterol levels, mid-blood pressure (mean of systolic and diastolic blood pressure), and fasting glucose levels. The variables were scaled to mean 0 and standard deviation (SD) 1 (z-transformed) separately for sex and sampling occasion. Due to a skewed distribution, triglycerides were log transformed first. We calculated a composite MetS score by summing all scaled variables except total cholesterol, which could distort the score depending on the proportions of HDL and LDL/VLDL within the total cholesterol measurement. The MetS score was also scaled separately by sex and sampling occasion. As a sensitivity analysis, we defined a dichotomous MetS variable according to the International Diabetes Federation criteria of obesity (BMI ≥ 30 kg/m^2^) and at least two of elevated triglyceride levels (≥1.7 mmol/l or lipid-lowering medication), hypertension (SBT ≥ 130 mm Hg or DBT ≥ 85 mm Hg or antihypertension medication), and elevated fasting glucose levels (≥5.6 mmol/l or self-reported diabetes).

### 2.3. Protein Biomarkers

All 276 samples were analyzed simultaneously for 178 unique protein biomarkers on two predesigned Proseek Multiplex® immunoassay panels (Olink Proteomics, Uppsala, Sweden) related to inflammation and cancer (all proteins are listed in Supplementary Table S1 available online at https://doi.org/10.1155/2017/4803156). Processing, output data quality check, and normalization were performed by Olink Proteomics. All data were delivered as Normalized Protein eXpression (NPX) values on a log2 scale. The log2 NPX values were scaled to mean 1 and SD 1 before data analysis, to facilitate comparisons between protein associations. Data values below the level of detection (LOD) were removed from the dataset. Proteins with <50% missing values (IL-20RA, IL-2RB, IL-1-alpha, IL-2, TSLP, IL-10RA, IL-22.RA1, IL-24, IL-13, ARTN, TNF, IL-20, IL-33, IFN-gamma, IL-4, LIF, NRTN, and IL-5) were excluded, leaving 160 proteins for further analysis.

### 2.4. Statistics

All computations were conducted in R v.3.3.1 (R Foundation for Statistical Computing, Vienna, Austria).

Associations between protein markers and MetS were determined by fitting linear mixed models for each protein using the lme function in the lmer R-package. The mixed models included participant as a random factor (random intercept) and MetS and covariates as fixed factors. Two models were fitted for each protein, one including MetS score and one including each individual MetS component. Other covariates adjusted for the models were CRC status (case, control), age (continuous), sex (male, female), physical activity (5-level scale from never to >3 times/week), smoking (non-, current, and ex-smoker), and level of education (elementary school, upper secondary school, and university). The contribution of MetS and its components to protein variance was tested by an analysis of variance approach using the anova.lme function. For evaluation of variation in protein levels within and between individuals, we calculated intraclass correlations (ICC), defined as the proportion of total variance due to variation between individuals, using the variance estimates from the mixed models. We also calculated variance explained by fixed factors (*R*^2^_m_) and variance explained by fixed and random factors (*R*^2^_c_) using the RsqGLMM function in the MuMIn package. Model assumptions were evaluated by visually inspecting the Pearson standardized residuals. Outliers, defined as standardized residuals >3, were excluded separately for each protein. Coefficients from the mixed models are interpreted as SD change in protein levels per 1SD change in MetS variable.

We assessed MetS and lifestyle-adjusted associations between the MetS-associated proteins by calculating partial Spearman's correlations on the estimated residuals from the mixed models using the core function on pairwise complete observations.

All *P* values were adjusted for multiple testing using the Bonferroni method. *P* values below 0.05 were considered significant.

We also examined MetS and the MetS-associated proteins in relation to CRC risk using conditional logistic regression models stratified on the matched case sets. Odds ratios (ORs) were estimated per 1SD change in the MetS variables. To evaluate whether associations differed depending on the follow-up time from sampling to CRC diagnosis, we tested for an interaction between sampling time point, MetS, and the protein variables.

## 3. Results

### 3.1. Participant Characteristics

Characteristics of the participants at the first and second sampling occasion are presented in Table 1(a), and characteristics stratified by case status in Table 1(b). Median age at first sampling was 49.9 years. Systolic blood pressure and fasting plasma glucose increased slightly over time. BMI also increased modestly with time, with more individuals categorized as obese at the second sampling occasion. The prevalence of MetS increased from 9% at the first sampling occasion to 17% at the second (Table 1(a)). BMI had a larger proportion of total variance between, and less within, participants compared to the other MetS components (ICC = 0.86, Supplementary Figure S1). Total cholesterol was higher in cases compared to controls (*P* = 0.03, Table 1(b)). There were no other large differences in MetS components, MetS score, or MetS prevalence between CRC cases and controls. The same pattern was seen at the first sampling occasion.

### 3.2. Protein Levels

**Figure 1:**
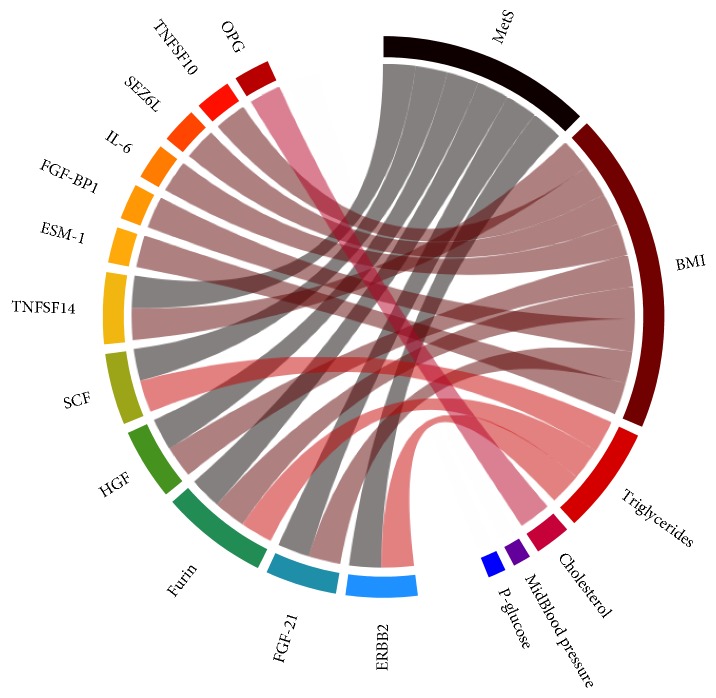
Significant associations between metabolic syndrome (MetS) and its components and each protein. Connections illustrate significant contributions to protein variance (Bonferroni corrected *P* value < 0.05).

**Figure 2:**
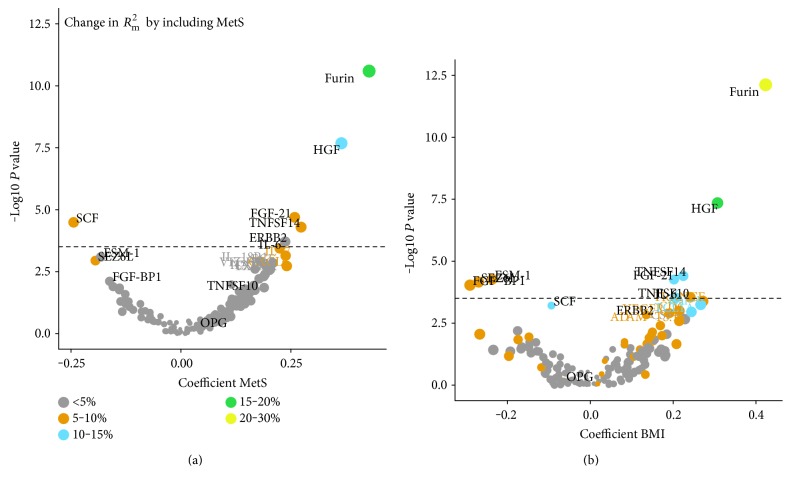
Volcano plots for metabolic syndrome (MetS) (a) and BMI (b). The dashed line indicates the Bonferroni-adjusted significance threshold. Coefficients are interpreted as SD change in protein levels by 1SD change in MetS score and BMI, respectively. *R*^2^_m_ is the proportion of variance explained by the included fixed factors.

Out of 160 proteins that passed quality control, six were associated with the MetS score: three (TNFSF14, HGF, and FGF-21) with MetS score and BMI, two (SCF and ERBB2) with MetS score and triglyceride levels, and one (Furin) with MetS score, BMI, and triglyceride levels. An additional five proteins were associated with BMI alone (TNFSF10, SEZ6L, IL-6, FGF-BP1, and ESM-1), and one (OPG) was associated with total cholesterol (Figure 1, Table 2). The direction of the significant associations was inverse for SCF, ESM-1, SEZ6L, and FGF-BP1 and positive for OPG, TNFSF10, IL-6, ERBB2, TNFSF14, FGF-21, HGF, and Furin (Figure 2, Supplementary Figure S2).

**Figure 3:**
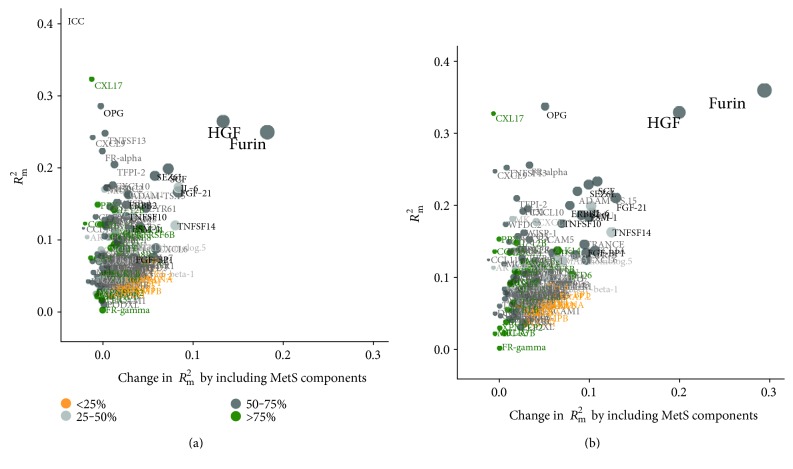
Contribution to variance explained by metabolic syndrome (MetS) and other included covariates. (a) Variance explained by including MetS (*x*-axis). (b) Variance explained by including MetS components. The proteins are color coded according to ICC, that is, proportion of interindividual variance to total variance. Proteins with high ICC vary less within and more between participants, whereas proteins with low ICC vary less between and more within participants. Proteins in black were significantly associated with MetS or any MetS component.

The proportion of variance in protein levels explained by the fixed factors in our models, *R*^2^_m_, varied between 0 and 35% (Figure 3). The proportion of variance attributable to MetS was 5–30% for the proteins identified. IL-6, FGF-21, and TNFSF14 varied more within participants between the measurements compared with the other significant proteins (ICC < 0.50, Supplementary Figure S3), whereas the intraindividual variation between sampling occasions was lower for SEZ6L, TNFSF10, and FGF-BP1 (ICC: 0.69–0.71).

Furin and HGF showed the strongest associations with MetS. The relations were driven largely by BMI, with 20–30 percentage points of the variation explained by the fixed factors attributed to BMI (Figure 3). OPG was significantly associated with total cholesterol, yet only a small proportion (5 percentage points) of the variance explained by the included fixed factors was attributable to MetS components. Instead, a large part (25%) of the variance in OPG was explained by age. Inthe sensitivity analyses, using predefined cut-offs to define MetS and MetS components gave similar results but with fewer significant protein associations (data not shown).

Partial correlations between the MetS-associated proteins are presented in Supplementary Figure S4. Almost all correlations were positive. Two clusters of more correlated proteins were present: cluster 1: SCF, OPG, ERBB2, SEZ6L, ESM-1, and FGF-BP1; cluster 2: HGF, TNFSF14, Furin, IL-6, FGF-21, and TNFSF10.

### 3.3. MetS, Protein Levels, and CRC Risk

**Figure 4:**
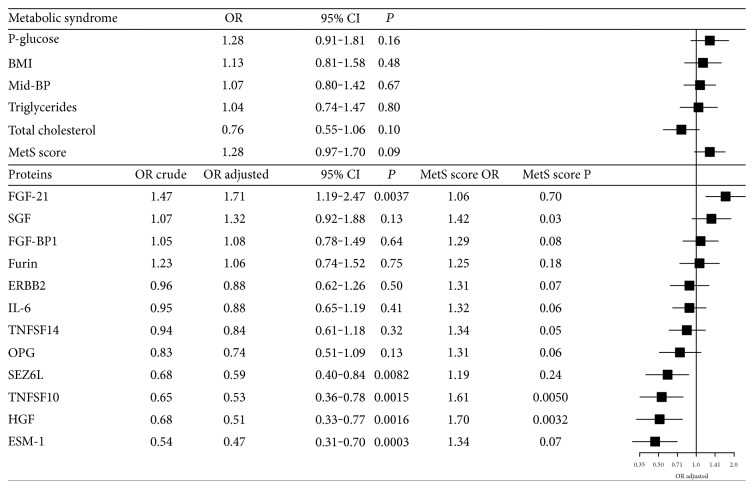
Associations between metabolic syndrome (MetS) score, MetS components, protein levels, and CRC risk. All odds ratios (ORs) were calculated using conditional logistic regression, stratified for the case-sets. For MetS, odds ratios (ORs) were adjusted for smoking status and education level. For protein models, ORs are adjusted for smoking status and education level. ORs adjusted are additionally adjusted for the MetS score. MetS score OR and MetS score P are ORs and corresponding *P* values for the MetS score in the protein models.

None of the MetS components was significantly associated with CRC risk in conditional logistic regression models adjusting for age, sex, and sampling date by case-set stratification, and additionally by smoking status and educational level by regression (Figure 4). MetS score was associated with an insignificant increased risk of CRC (OR per 1SD increase in MetS score: 1.28, 95% CI: 0.97–1.70). For the 12 proteins significantly associated with MetS and/or its components, five were associated with CRC risk. Higher levels of FGF-21, which in our dataset were directly associated with MetS score and BMI, were associated with an increased CRC risk (OR per 1SD increase in protein levels: 1.71, 95% CI: 1.19–2.47). Higher levels of SEZ6L, TNFSF10, HGF, and ESM-1 were associated with a lower CRC risk (ORs per 1SD increase in protein levels: 0.59 to 0.39). Most protein risk estimates were enlarged by including MetS score in the models. The OR for MetS score was markedly enlarged when including SCF, TNFSF10, and HGF and attenuated when including FGF-21 and SEZ6L (Figure 4). Similar changes in ORs were seen for BMI. There were no significant interactions between MetS and the proteins, or between MetS or MetS-related proteins, and sampling time point.

## 4. Discussion

Metabolic syndrome is becoming increasingly common, and many studies indicate a direct association between MetS and the risk of developing CRC and other forms of cancer, likely driven, at least in part, by body composition and inflammation [13, 14]. In order to investigate the hypothesis that inflammation is the driving factor between MetS and CRC, we evaluated 160 unique protein biomarkers, known to be related to cancer or inflammation, in repeated samples from 138 individuals (of which half developed CRC within 5 years after the second sampling occasion). Twelve proteins were associated with at least one MetS component, six of which were also associated with MetS score. One of the six, FGF-21, was positively associated with CRC risk.

Interestingly, five of the 12 proteins identified were associated with BMI only, all of which were included in the predefined oncology protein marker panel due to a potential relation to cancer (with or without an inflammatory connection). However, of the six proteins associated with MetS score, there was an equal distribution between inflammatory and cancer proteins. Thus, body composition likely contributes to cancer development not only through chronic inflammation but also through other pathways.

Of the MetS-associated proteins, only one, which was also associated with BMI, was positively associated with CRC risk, FGF-21 (fibroblast growth factor 21). Inclusion of MetS strengthened the association between FGF-21 and CRC risk and attenuated the association between MetS and CRC risk, suggesting a mediating effect. Increases in BMI and MetS score contributed to a significant amount of the FGF-21 protein level variation, and it was the protein with the largest change in level per MetS score increase. Consistent with our observations, the associations between FGF-21, BMI, and MetS have been previously described [15, 16] and appear to be robust in plasma or serum samples. FGF-21 is part of the family of fibroblast growth factors, which includes 18 mammalian proteins (FGF1-FGF10 and FGF16-FGF23) [17]. However, FGF-21 is not a clear-cut growth factor but serves as a metabolic hormone as it lacks the FGF-heparin-binding domain and therefore can diffuse away from its tissue of origin [18, 19]. FGF-21 is expressed in a wide variety of tissues [17] and mediates signaling by binding to the tyrosine kinase FGF-receptors. One of the main functions of FGF-21 appears to be the regulation of metabolic function and stimulation of glucose uptake [20]. It has been shown to increase in patients with T2DM [21]. More recently, the possibility of using FGF-21 as a prognostic or diagnostic cancer marker has been raised and evaluated for renal cancer, with promising results [22]. To the best of our knowledge, FGF-21 has not previously been evaluated as a biomarker for CRC risk. Our results indicate that it might be suitable for this purpose. However, FGF-21 levels were subject to a fairly high intraindividual variation (0.46), meaning variation over time is common. Intraindividual variation would need to be taken into consideration for all future applications including FGF-21 as a potential biomarker [23].

Two proteins were strongly associated with both MetS and BMI, namely, HGF (hepatocyte growth factor) and Furin. Both of these proteins have been previously implicated in MetS [24, 25]. HGF is a cytokine secreted from adipocyte tissue, known to increase with hypertension and obesity and most likely regulated by genetic factors [26]. In the present study, HGF was positively related to both MetS score and BMI but inversely associated with CRC risk, an effect that was strengthened by adjusting for MetS score. This contradicts a previous study in which elevated serum HGF levels were proposed as a biomarker for tumor progression and suggested to enhance angiogenesis and tumor cell invasion [27]. Furin belongs to the proprotein convertase family and processes inactive proteins into their active forms [28]. It is the most studied protein in this family, and its function and expression have been investigated in relation to several types of cancer. Furin's role in protein activation makes it important for tumor progression and metastasis. Inhibitors of Furin activity are therefore potential targets for cancer therapy [29]. At the same time, overexpression of Furin has been linked to tumor suppression and better prognosis in hepatocellular carcinoma [30]. We found no significant association between Furin and CRC in our dataset, possibly due to the prospective study design, with plasma samples collected prior to CRC diagnosis. However, the null relationship was apparent even at the second sampling occasion, at which cases were more likely to have a premalignant or malignant process in the colorectum.

The protein most strongly associated with CRC risk was ESM-1 (endothelial cell-specific molecule-1), which was inversely associated with both BMI and CRC in our dataset but not associated with MetS. ESM-1 has previously been shown to be overexpressed in CRC patients and associated with poor prognosis [31]. The fact that increased levels in our dataset were indicative of reduced CRC risk shows the difficulty in translating markers identified at diagnosis to prospective samples. ESM-1 does appear to have an active role in CRC by regulating growth and metastasis and may be useful as a therapeutic target [32], but further investigation of the mechanisms behind the inverse association we observed using prediagnostic samples is warranted.

One other protein, SEZ6L (seizure 6-like protein), was also inversely associated with both BMI and CRC. It controls synaptic connectivity and motor coordination and is also a substrate for the *β*-secretase BACE1, which is highly expressed in the nervous system and an important drug target in Alzheimer's disease [33]. Although it has not previously been implicated in CRC, one study found an association between a genetic polymorphism in the SEZ6L gene and increased risk of lung cancer [34].

Weaknesses of our study include lack of a central obesity measurement (substituted with BMI) and HDL cholesterol measurements. Total cholesterol was evaluated in relation to protein measurements but not included in the MetS score definition because of the conflicting roles of HDL and LDL, both of which contribute to total cholesterol. In addition, CRP (c-reactive protein), an established and important marker of inflammation previously connected to MetS [35], was not included in the Olink inflammation panel, and TNF-alpha (a well-known marker of inflammation and characteristic of MetS [8]) was omitted due to missing values. Hence, neither of these were addressed in our study.

Major strengths of the study included the use of high-quality blood samples collected prospectively with respect to CRC diagnosis, with all participants fasting for at least eight hours prior to sampling, and with no previous thaw-freeze cycles. The VIP cohort also provided a unique opportunity to use repeated samples from both cases and time-matched controls, allowing us to account for intra-individual variation. Finally, an important strength of the study was the large number of protein biomarkers evaluated simultaneously using a highly sensitive platform.

## 5. Conclusions

In our study MetS does not, in itself, appear to contribute to the inflammation cancer-connection. MetS score was associated with six different proteins in our investigation. However, all were also associated with BMI and/or triglyceride levels. Of the individual MetS components assessed, BMI displayed by far the strongest associations with inflammatory and cancer biomarkers. Although external replication is needed, our data indicate that the relationship between MetS and CRC risk is likely driven primarily by excess body fat, acting through both pro-inflammation and other pro-carcinogenic mechanisms.

## Conflicts of Interest

The authors declare that they have no conflicts of interest.

## Supplementary Material

Figure S1: MetS and its components per individual over time. Figure S2: Levels of MetS associated proteins (log2) per individual over time. Figure S3: Direction of protein-MetS associations. Figure S4: Protein-protein associations of MetS associated proteins. Table S1: Proteins included in Proseek Multiplex® immunoassay panels.

## Figures and Tables

**(a):** 

Characteristics	Sample 1 (*n* = 138^a^)	Sample 2 (*n* = 138^a^)	*P* ^b^
	Median (range)	Median (range)	
Age	49.9 (30.0–52.4)	59.9 (40.0–60.5)	—
Height	173 (157–195)	172 (155–196)	<0.001
Weight	76.5 (51–128)	80.0 (51–139)	<0.001
Triglycerides, mmol/l	1.0 (0.4-5.2)	1.1 (0.5–3.8)	0.98
Total cholesterol, mmol/l	5.5 (3.5–9.5)	5.5 (3.4–10.1)	0.18
Diastolic bp	79.0 (60.0–120.0)	80.0 (60.0–110.0)	0.12
Systolic bp	120.0 (94.0–180.0)	126.0 (90.0–179.0)	0.04
Fasting plasma glucose, mmol/l	5.4 (4.0–6.9)	5.5 (1.0–13.1)	0.003
BMI	25.3 (18.8–41.3)	26.0 (18.4–44.9)	<0.001
Categorized BMI (*N*, (%))	—	—	0.08
<18.5 (underweight)	0	1 (1)	—
18.5–24.9 (normal)	62 (45)	52 (38)	—
25–29.9 (overweight)	60 (43)	56 (41)	—
30+ (obese)	16 (16)	29 (21)	—
MetS (yes, %)	12 (9)	24 (17)	0.05

^a^Number of samples.

^b^Paired *t*-tests for differences in continuous variables between measurements 1 and 2. Chi-square test or Fisher's exact test (if expected cell count is below 5) for differences in categorical variables.

**(b):** 

Characteristics	Case (*n* = 69)	Control (*n* = 69)	*P* ^b^
	Median (range)	Median (range)	
Age (at sampling occasion)	59.9 (40.3–60.4)	59.9 (40.0–60.5)	—
Height	172.5 (156–196)	172 (155–191)	0.99
Weight	81.0 (56–114)	78.0 (51–139)	0.95
Triglycerides, mmol/l	1.1 (0.6–3.5)	1.1 (0.5–3.8)	0.99
Total cholesterol, mmol/l	5.4 (3.8–7.6)	5.6 (3.4–10.1)	0.03
Diastolic bp	82.0 (62.0–105.0)	80.0 (60.0–110.0)	0.14
Systolic bp	126.0 (90.0–163.0)	126.0 (90.0–179.0)	0.80
Fasting plasma glucose, mmol/l	5.7 (1.0–11.1)	5.5 (4.1–13.1)	0.73
BMI	26.1 (21.0–36.0)	26.0 (18.4–44.9)	0.82
Categorized BMI (*N*, (%))	—	—	0.12
<18.5 (underweight)	0	1 (1)	—
18.5–24.9 (normal)	22 (32)	30 (43)	—
25–29.9 (overweight)	34 (50)	22 (32)	—
30+ (obese)	12 (18)	16 (23)	—
MetS score	−0.02 (−0.60–0.54)	−0.06 (−0.80–0.51)	0.26
MetS (yes, %)	9 (13)	15 (22)	—

All data refer to characteristics at the second sampling as depicted in Table 1(a).

^b^Paired *t*-tests for differences in continuous variables between matched cases and controls. Chi-square test or Fisher's exact test (if expected cell count is below 5) for differences in categorical variables.

**Table 2:** List of proteins significantly associated with MetS score or one of its components.

Protein name	UniProt number	Associated with	Direction of association	Original Olink panel
IL-6	P05231	BMI	Positive	Inflammation panel and oncology panel
TNFSF10	P50591	BMI	Positive	Inflammation panel and oncology panel
SEZ6L	Q9BYH1	BMI	Negative	Oncology panel
FGF-BP1	Q14512	BMI	Negative	Oncology panel
ESM-1	Q9NQ30	BMI	Negative	Oncology panel
FGF-21	Q9NSA1	MetS and BMI	Positive	Inflammation panel
TNFSF14	O43557	MetS and BMI	Positive	Inflammation panel
HGF	P14210	MetS and BMI	Positive	Inflammation panel and oncology panel
Furin	P09958	MetS, BMI, and triglycerides	Positive	Oncology panel
ERBB2	P04626	MetS and triglycerides	Positive	Oncology panel
SCF	P21583	MetS and triglycerides	Negative	Inflammation panel and oncology panel
OPG	O00300	Total cholesterol	Positive	Inflammation panel
