# Supplementary material for: The Metabolic Syndrome, Inflammation, and Colorectal Cancer Risk: An Evaluation of Large Panels of Plasma Protein Markers Using Repeated, Prediagnostic Samples
Source: Mediators Inflamm. 2017 Mar 22;2017:4803156. doi: 10.1155/2017/4803156 (PMC5381203; doi:10.1155/2017/4803156)
Supplement: Supplementary file 1 — Figure S1: MetS and its components per individual over time. Figure S2: Levels of MetS associated proteins (log2) per individual over time. Figure S3: Direction of protein-MetS associations. Figure S4: Protein-protein associations of MetS associated proteins. Table S1: Proteins included in Proseek Multiplex® immunoassay panels. [file 4803156.f1.pdf]

Supplemental Material

The metabolic syndrome, inflammation and colorectal cancer risk; an evaluation of large panels of plasma protein markers using repeated, pre-diagnostic samples

Sophia Harlid, Robin Myte, Bethany Van Guelpen

Table of Contents: **Page**

Table of Contents:	Page
Figure S1 (MetS and its components per individual over time)	2
Figure S2 (Levels of MetS associated proteins (log2) per individual over time)	3
Figure S3 (Direction of protein-MetS associations)	4
Figure S4 (Protein-protein associations of MetS associated proteins)	5
Table S1 (Proteins included in Proseek Multiplex® immunoassay panels)	6-10

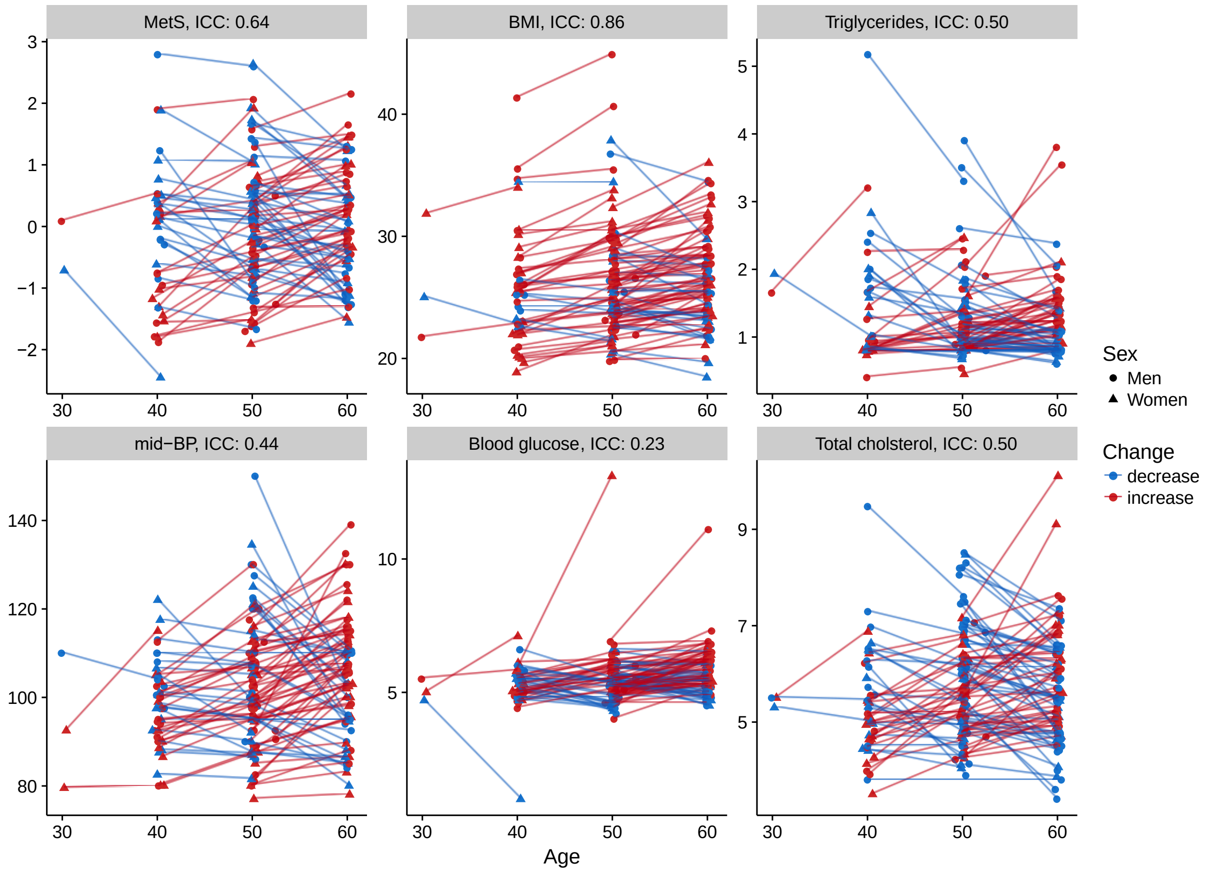

Figure S1. Metabolic syndrome (MetS) and its components in the study participants over time. ICC: Intraclass correlation coefficient, i.e., proportion of total variance due to variation between individuals.

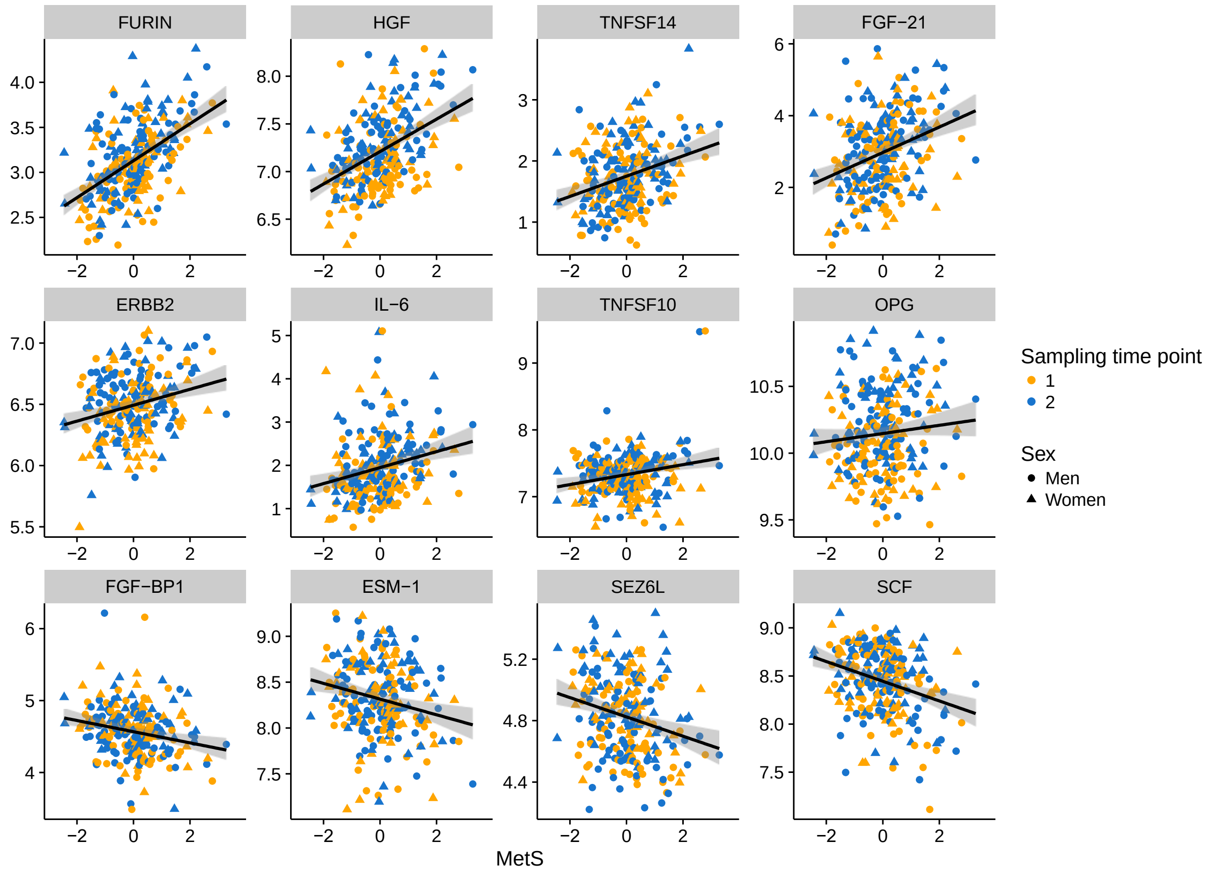

Figure S2. Direction of protein-metabolic syndrome (MetS) associations. Protein levels (log2) plotted against MetS score, and regression lines with error bounds plotted without random intercepts for simplicity. The proteins are sorted according to size and direction of regression coefficient.

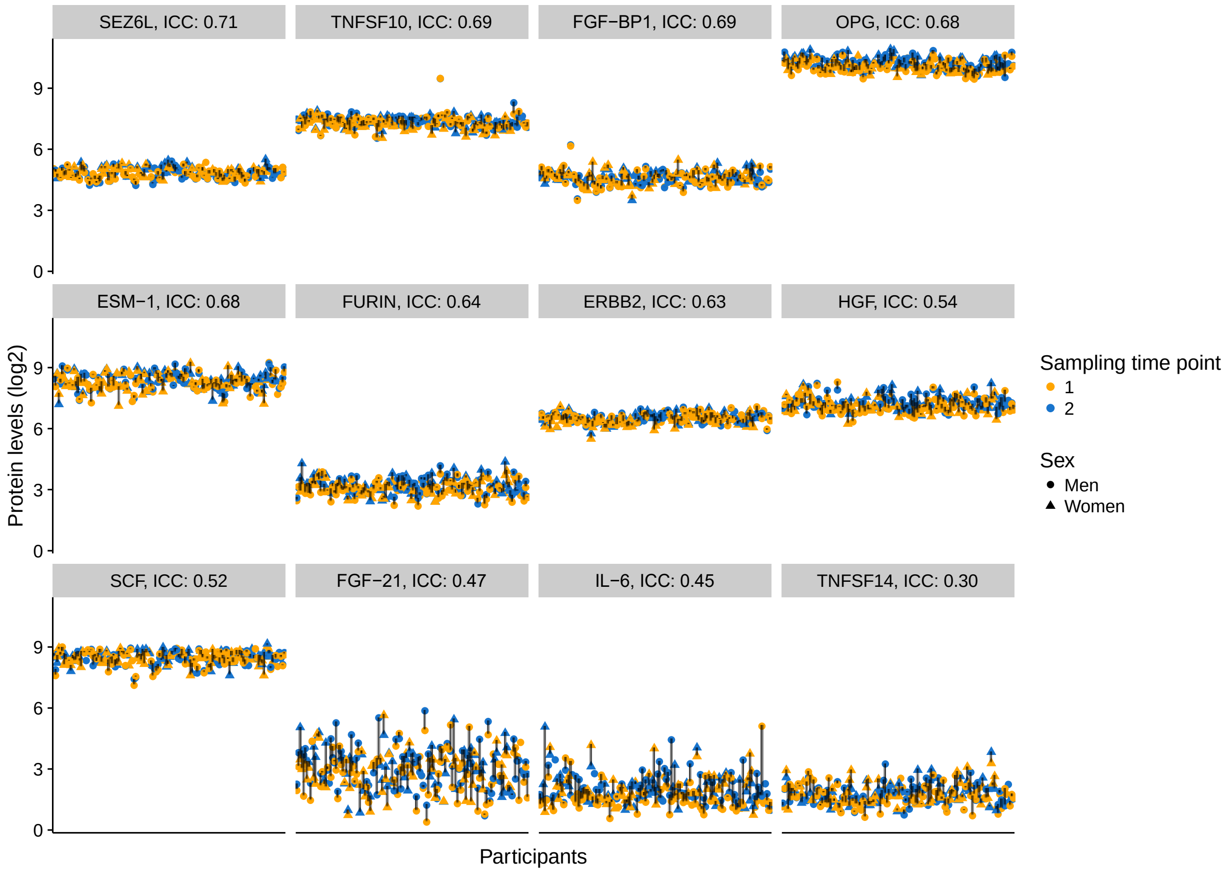

Figure S3. Levels of metabolic syndrome (MetS) associated proteins (log2) per participant by sampling time point. The proteins are sorted according to ICC **(**ICC: Intra-class correlation coefficient, i.e., proportion of total variance due to variation between individuals.)

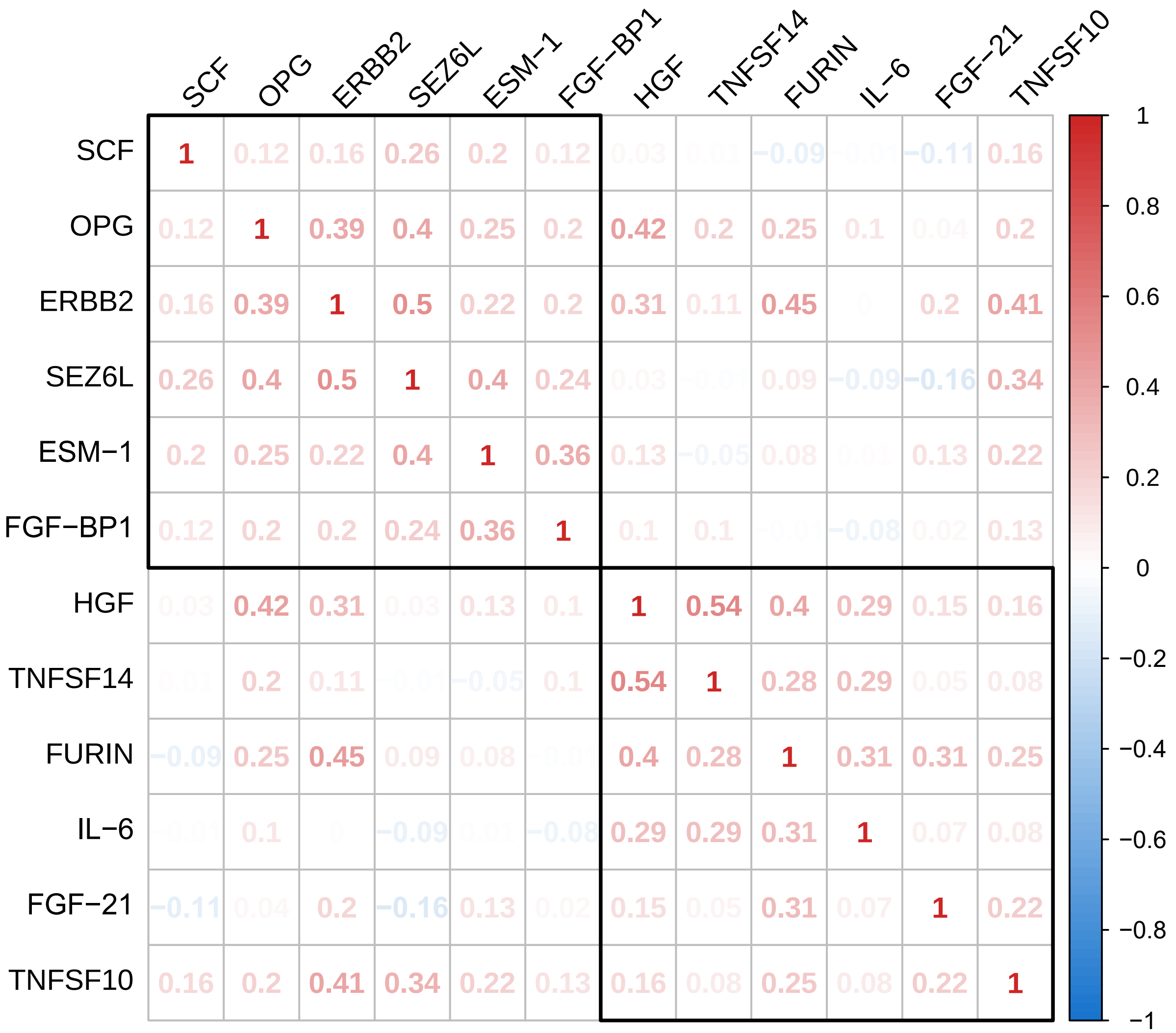

Figure S4. Partial correlations between metabolic syndrome (MetS) -associated proteins. Partial correlations were calculated by estimating Spearman's correlations on the standardized residuals from protein mixed models adjusting for age, sex, MetS, physical activity, smoking and education level. The black squares represent the two major clusters from a hierarchical cluster analysis on the correlations (using Euclidean distance with average linkage).

Table S1. Proteins included in Proseek Multiplex® immunoassay panels

Protein	UniProt No	Immunoassay panel	Comment
Hepatocyte growth factor (HGF)	P14210	Both	
Interleukin-6 (IL-6)	P05231	Both	
Stem cell factor (SCF)	P21583	Both	Excluded from one panel
TNF-related apoptosis-inducing ligand (TRAIL)	P50591	Both	Excluded from one panel
Transforming growth factor alpha (TGF-alpha)	P01135	Both	
Vascular endothelial growth factor A (VEGF-A)	P15692	Both	
Adenosine Deaminase (ADA)	P00813	Inflammation	
Artemin (ARTN)	Q5T4W7	Inflammation	Excluded (>50% missing)
Axin-1 (AXIN1)	O15169	Inflammation	
Beta-nerve growth factor (Beta-NGF) P01138	P01138	Inflammation	
Brain-derived neurotrophic factor (BDNF)	P23560	Inflammation	
Caspase 8 (CASP-8)	Q14790	Inflammation	
C-C motif chemokine 19 (CCL19)	Q99731	Inflammation	
C-C motif chemokine 20 (CCL20)	P78556	Inflammation	
C-C motif chemokine 23 (CCL23)	P55773	Inflammation	
C-C motif chemokine 25 (CCL25)	O15444	Inflammation	
C-C motif chemokine 28 (CCL28)	Q9NRJ3	Inflammation	
C-C motif chemokine 4 (CCL4)	P13236	Inflammation	
CD40L receptor (CD40)	P25942	Inflammation	
CUB domain-containing protein 1 (CDCP1)	Q9H5V8	Inflammation	
C-X-C motif chemokine 1 (CXCL1)	P09341	Inflammation	
C-X-C motif chemokine 10 (CXCL10)	P02778	Inflammation	
C-X-C motif chemokine 11 (CXCL11)	O14625	Inflammation	
C-X-C motif chemokine 5 (CXCL5)	P42830	Inflammation	
C-X-C motif chemokine 6 (CXCL6)	P80162	Inflammation	
C-X-C motif chemokine 9 (CXCL9)	Q07325	Inflammation	
Cystatin D (CST5)	P28325	Inflammation	
Delta and Notch-like epidermal growth factor-related recep (DNER)	Q8NFT8	Inflammation	
Eotaxin-1 (CCL11)	P51671	Inflammation	
Eukaryotic translation initiation factor 4E-binding protein 1 (4E-BP1)	Q13541	Inflammation	
Fibroblast growth factor 19 (FGF-19)	O95750	Inflammation	
Fibroblast growth factor 21 (FGF-21)	Q9NSA1	Inflammation	
Fibroblast growth factor 23 (FGF-23)	Q9GZV9	Inflammation	
Fibroblast growth factor 5 (FGF-5)	Q8NF90	Inflammation	
Fms-related tyrosine kinase 3 ligand (Flt3L)	P49771	Inflammation	
Fractalkine (CX3CL1)	P78423	Inflammation	
Glial cell line-derived neurotrophic factor (hGDNF)	P39905	Inflammation	

Table continues on next page

Table S1. Cont.

Protein	UniProt No	Immunoassay panel	Comment
Interferon gamma (IFN-gamma)	P01579	Inflammation	
Interleukin-1 alpha (IL-1 alpha)	P01583	Inflammation	Excluded (>50% missing)
Interleukin-10 (IL-10)	P22301	Inflammation	
Interleukin-10 receptor subunit alpha (IL-10RA)	Q13651	Inflammation	Excluded (>50% missing)
Interleukin-10 receptor subunit beta (IL-10RB)	Q08334	Inflammation	
Interleukin-12 subunit beta (IL-12B)	P29460	Inflammation	
Interleukin-13 (IL-13)	P35225	Inflammation	Excluded (>50% missing)
Interleukin-15 receptor subunit alpha (IL-15RA)	Q13261	Inflammation	
Interleukin-17A (IL-17A)	Q16552	Inflammation	
Interleukin-17C (IL-17C)	Q9P0M4	Inflammation	
Interleukin-18 (IL-18)	Q14116	Inflammation	
Interleukin-18 receptor 1 (IL-18R1)	Q13478	Inflammation	
Interleukin-2 (IL-2)	P60568	Inflammation	Excluded (>50% missing)
Interleukin-2 receptor subunit beta (IL-2RB)	P14784	Inflammation	Excluded (>50% missing)
Interleukin-20 (IL-20)	Q9NYY1	Inflammation	Excluded (>50% missing)
Interleukin-20 receptor subunit alpha (IL-20RA)	Q9UHF4	Inflammation	Excluded (>50% missing)
Interleukin-22 receptor subunit alpha-1 (IL-22 RA1)	Q8N6P7	Inflammation	Excluded (>50% missing)
Interleukin-24 (IL-24)	Q13007	Inflammation	Excluded (>50% missing)
Interleukin-33 (IL-33)	O95760	Inflammation	Excluded (>50% missing)
Interleukin-4 (IL-4)	P05112	Inflammation	Excluded (>50% missing)
Interleukin-5 (IL-5)	P05113	Inflammation	Excluded (>50% missing)
Interleukin-7 (IL-7)	P13232	Inflammation	
Interleukin-8 (IL-8)	P10145	Inflammation	
Latency-associated peptide transforming growth factor beta 1 (LAP TGF-beta-1)	P01137	Inflammation	
Leukemia inhibitory factor (LIF)	P15018	Inflammation	Excluded (>50% missing)
Leukemia inhibitory factor receptor (LIF-R)	P42702	Inflammation	
Macrophage colony-stimulating factor 1 (CSF-1)	P09603	Inflammation	
Macrophage inflammatory protein 1-alpha (MIP-1 alpha)	P10147	Inflammation	Excluded (>50% missing)
Matrix metalloproteinase-1 (MMP-1)	P03956	Inflammation	
Matrix metalloproteinase-10 (MMP-10)	P09238	Inflammation	
Monocyte chemotactic protein 1 (MCP-1)	P13500	Inflammation	
Monocyte chemotactic protein 2 (MCP-2)	P80075	Inflammation	
Monocyte chemotactic protein 3 (MCP-3)	P80098	Inflammation	
Monocyte chemotactic protein 4 (MCP-4)	Q99616	Inflammation	
Natural killer cell receptor 2B4 (CD244)	Q9BZW8	Inflammation	
Neurotrophin-3 (NT-3)	P20783	Inflammation	
Neurturin (NRTN)	Q99748	Inflammation	Excluded (>50% missing)

Table continues on next page

Table S1. Cont.

Protein	UniProt No	Immunoassay panel	Comment
Oncostatin-M (OSM)	P13725	Inflammation	
Osteoprotegerin (OPG)	O00300	Inflammation	
Programmed cell death 1 ligand 1 (PD-L1)	Q9NZQ7	Inflammation	
Protein S100-A12 (EN-RAGE)	P80511	Inflammation	
Signaling lymphocytic activation molecule (SLAMF1)	Q13291	Inflammation	
SIR2-like protein 2 (SIRT2)	Q8IXJ6	Inflammation	
STAM-binding protein (STAMPB)	O95630	Inflammation	
Sulfotransferase 1A1 (ST1A1)	P50225	Inflammation	
T cell surface glycoprotein CD6 isoform (CD6)	Q8WWJ7	Inflammation	
T-cell surface glycoprotein CD5 (CD5)	P06127	Inflammation	
Thymic stromal lymphopoietin (TSLP)	Q969D9	Inflammation	Excluded (>50% missing)
TNF-beta (TNFB)	P01374	Inflammation	
TNF-related activation-induced cytokine (TRANCE)	O14788	Inflammation	
Tumor necrosis factor (Ligand) superfamily, member 12 (TWEAK)	Q4ACW9	Inflammation	
Tumor necrosis factor (TNF)	P01375	Inflammation	Excluded (>50% missing)
Tumor necrosis factor ligand superfamily member 14 (TNFSF14)	O43557	Inflammation	
Tumor necrosis factor receptor superfamily member 9 (TNFRSF9)	Q07011	Inflammation	
Urokinase-type plasminogen activator (uPA)	P00749	Inflammation	
5'-nucleotidase (5'-NT)	P21589	Oncology II	
A disintegrin and metalloproteinase with thrombospondin motifs 15 (ADAM-TS 15)	Q8TE58	Oncology II	
A/B (MIC-A/B)	Q29980	Oncology II	
AlPha-taxilin (TXLNA)	P40222	Oncology II	
AmPhiregulin (AR)	P15514	Oncology II	
Annexin A1 (ANXA1)	P04083	Oncology II	
Carbonic anhydrase 9 (CA9)	Q16790	Oncology II	
Carboxypeptidase E (CPE)	P16870	Oncology II	
Carcinoembryonic antigen-related celladhesion molecule 1 (CEACAM1)	P13688	Oncology II	
Carcinoembryonic antigen-related celladhesion molecule 5 (CEACAM5)	P06731	Oncology II	
Cathepsin L2 (CTSV)	O60911	Oncology II	
CD160 antigen (CD160)	O95971	Oncology II	
CD27 antigen (CD27)	P26842	Oncology II	
CD48 antigen (CD48)	P09326	Oncology II	
CD70 antigen (CD70)	P32970	Oncology II	
Cornulin (CRNN)	Q9UBG3	Oncology II	
C-type lectin domain family 4 member K(CD207)	Q9UJ71	Oncology II	
C-X-C motif chemokine 13 (CXCL13)	O43927	Oncology II	
Cyclin-dependent kinase inhibitor 1 (CDKN1A)	P38936	Oncology II	Excluded (>50% missing)

Table continues on next page

Table S1. Cont.

Protein	UniProt No	Immunoassay panel	Comment
Delta-like protein 1 (DLL1)	O00548	Oncology II	
Disintegrin and metalloproteinase domain-containing protein (ADAM8)	P78325	Oncology II	
Endothelial cell-specific molecule 1 (ESM-1)	Q9NQ30	Oncology II	
EPhrin type-A receptor 2 (EPH2)	P29317	Oncology II	
FAS-associated death domain protein (FADD)	Q13158	Oncology II	
Fc receptor-like B (FCRLB)	Q6BAA4	Oncology II	
Fibroblast growth factor-binding Protein 1(FGF-BP1)	Q14512	Oncology II	
Folate receptor alpha (FR-alpha)	P15328	Oncology II	
Folate receptor gamma (FR-gamma)	P41439	Oncology II	
Furin (FURIN)	P09958	Oncology II	
Galectin-1 (Gal-1)	P09382	Oncology II	
Glypican-1 (GPC1)	P35052	Oncology II	
Granzyme B (GZMB)	P10144	Oncology II	
Granzyme H (GZMH)	P20718	Oncology II	
ICOS ligand (ICOSLG)	O75144	Oncology II	
Insulin-like growth factor 1 receptor (IGF1R)	P08069	Oncology II	Excluded (>50% missing)
Integrin alpha-V (ITGAV)	P06756	Oncology II	
Integrin beta-5 (ITGB5)	P18084	Oncology II	
Interferon gamma receptor 1 (IFN-gamma-R1)	P15260	Oncology II	
Kallikrein-11 (hK11)	Q9UBX7	Oncology II	
Kallikrein-13 (KLK13)	Q9UKR3	Oncology II	
Kallikrein-14 (hK14)	Q9P0G3	Oncology II	
Kallikrein-8 (hK8)	O60259	Oncology II	
Ly6/PLAUR domain-containing Protein 3 (LYPD3)	O95274	Oncology II	
Melanoma-derived growth regulatory Protein (MIA)	Q16674	Oncology II	
Mesothelin (MSLN)	Q13421	Oncology II	
Methionine aminoPeptidase 2 (MetAp2)	P50579	Oncology II	
MHC class I Polypeptide-related se Quence	Q29983	Oncology II	Excluded (>50% missing)
Midkine (Mk)	P21741	Oncology II	
Mothers against decapentaplegic homolog 5 (MAD homolog 5)	Q99717	Oncology II	
Mucin-16 (MUC-16)	Q8WXI7	Oncology II	
Nectin-4 (PVRL4)	Q96NY8	Oncology II	
Pancreatic prohormone (PPY)	P01298	Oncology II	
Podocalyxin (PODXL)	O00592	Oncology II	
Pro-epidermal growth factor (EGF)	P01133	Oncology II	
Protein CYR61 (CYR61)	O00622	Oncology II	
Protein S100-A11 (S100A11)	P31949	Oncology II	

Table continues on next page

Table S1. Cont.

Protein	UniProt No	Immunoassay panel	Comment
Protein S100-A4 (S100A4)	P26447	Oncology II	
Proto-oncogene tyrosine-protein kinasereceptor Ret (RET)	P07949	Oncology II	
Receptor tyrosineprotein kinase erbB-2(ERBB2)	P04626	Oncology II	
Receptor tyrosineprotein kinase erbB-3 (ERBB3)	P21860	Oncology II	
Receptor tyrosineprotein kinase erbB-4 (ERBB4)	Q15303	Oncology II	
R-spondin-3 (RSPO3)	Q9BXY4	Oncology II	
Secretory carrier-associated membraneprotein 3 (SCAMP3)	O14828	Oncology II	
Seizure 6-like protein (SEZ6L)	Q9BYH1	Oncology II	
SPARC (SPARC)	P09486	Oncology II	
Syndecan-1 (SYND1)	P18827	Oncology II	
T-cell leukemia / lymphoma Protein 1A (TCL1A)	P56279	Oncology II	
TGF-beta receptor type-2 (TGFR-2)	P37173	Oncology II	
Tissue factor pathway inhibitor 2 (TFP1-2)	P48307	Oncology II	
T-lymphocyte surface antigen Ly-9 (LY9)	Q9HBG7	Oncology II	
Toll-like receptor 3 (TLR3)	O15455	Oncology II	
Transmembrane glycoprotein NMB (GPNMB)	Q14956	Oncology II	
Tumor necrosis factor ligand superfamilymember 13 (TNFSF13)	O75888	Oncology II	
Tumor necrosis factor ligand superfamilymember 6 (FASLG)	P48023	Oncology II	
Tumor necrosis factor receptor superfamilymember 19 (TNFRSF19)	Q9NS68	Oncology II	
Tumor necrosis factor receptor superfamilymember 4 (TNFRSF4)	P43489	Oncology II	
Tumor necrosis factor receptor superfamilymember 6B (TNFRSF6B)	O95407	Oncology II	
Tyrosineprotein kinase ABL1 (ABL1)	P00519	Oncology II	
Tyrosineprotein kinase Lyn (LYN)	P07948	Oncology II	
Vascular endothelial growth factor receptor 2 (VEGFR-2)	P35968	Oncology II	
Vascular endothelial growth factor receptor 3 (VEGFR-3)	P35916	Oncology II	
VEGF-co regulated chemokine 1 (CXL17)	Q6UXB2	Oncology II	
Vimentin (VIM)	P08670	Oncology II	
WAP four-disulfide core domain protein 2 (WFDC2)	Q14508	Oncology II	
Wnt inhibitory factor 1 (WIF-1)	Q9Y5W5	Oncology II	
WNT1-inducible-signaling pathway protein 1 (WISP-1)	O95388	Oncology II	
Xaa-pro aminopeptidase 2 (XPNPEP2)	O43895	Oncology II	
